# Phosphodiesterase Type 4 Inhibitor Rolipram Improves Survival of Spiral Ganglion Neurons *In Vitro*

**DOI:** 10.1371/journal.pone.0092157

**Published:** 2014-03-18

**Authors:** Katharina Kranz, Athanasia Warnecke, Thomas Lenarz, Martin Durisin, Verena Scheper

**Affiliations:** Department of Otolaryngology, Hannover Medical School, Hannover, Germany; University of South Florida, United States of America

## Abstract

Sensorineural deafness is caused by damage of hair cells followed by degeneration of the spiral ganglion neurons and can be moderated by cochlear implants. However, the benefit of the cochlear implant depends on the excitability of the spiral ganglion neurons. Therefore, current research focuses on the identification of agents that will preserve their degeneration. In this project we investigated the neuroprotective effect of Rolipram as a promising agent to improve the viability of the auditory neurons. It is a pharmaceutical agent that acts by selective inhibition of the phosphodiesterase 4 leading to an increase in cyclic AMP. Different studies reported a neuroprotective effect of Rolipram. However, its significance for the survival of SGN has not been reported so far. Thus, we isolated spiral ganglion cells of neonatal rats for cultivation with different Rolipram concentrations and determined the neuronal survival rate. Furthermore, we examined immunocytologically distinct proteins that might be involved in the neuroprotective signalling pathway of Rolipram and determined endogenous BDNF by ELISA. When applied at a concentration of 0.1 nM, Rolipram improved the survival of SGN *in vitro*. According to previous studies, our immunocytological data showed that Rolipram application induces the phosphorylation and thereby activation of the transcription factor CREB. This activation can be mediated by the cAMP-PKA-signalling pathway as well as via ERK as a part of the MAP-kinase pathway. However, only in cultures pre-treated with BDNF, an endogenous increase of BDNF was detected. We conclude that Rolipram has the potential to improve the vitality of neonatal auditory nerve cells *in vitro*. Further investigations are necessary to prove the effect of Rolipram *in vivo* in the adult organism after lesion of the hair cells and insertion of cochlear implants.

## Introduction

The first auditory neurons, the spiral ganglion neurons (SGN), connect the hair cells of the auditory system with higher regions of the central auditory pathway. Interactions between inner hair cells and afferent fibres of the SGN occur in terms of signal transmission via glutamate release from depolarized hair cells and in terms of trophic support with growth factors like BDNF and NT3 delivered from the hair cells. Both kinds of interaction are essential for the maintenance of the homeostasis and functionality of the SGN. Therefore, age-, drug- and noise-induced damage and loss of the hair cells consequently causes a successive secondary degeneration of the SGN due to the absence of functional innervation and deprived neurotrophic support [1]. However, recent evidence shows that SGN degeneration in humans is not dependent on hair cell loss [2]. In addition, using a mouse model, the role of supporting cells in the maintenance of SGN was demonstrated [3]. One therapeutic measure to moderate or compensate the loss of the hair cells is the treatment with a cochlear implant that directly stimulates residual SGN. Although this is an ongoing controversial discussion, it is still believed that the benefit of such a cochlear implant strongly depends not only on the excitability of the SGN [4], [5], but also on the number of surviving neurons [5]–[7]. Thus, current research focuses on the preservation of unaffected and the regeneration of deprived SGN in addition to the electrical innervation provided by the cochlear implant. A potent approach to increase the viability of SGN *in vitro* and *in vivo* is the external application of BDNF [8]–[14].

In the cochlea, the protective effect of BDNF is primarily promoted by the activation of the high-affinity tyrosine kinase receptor B (TrkB) [15]. TrkB signals via an intracellular cascade that is connected to the extracellular signal-regulated kinase (ERK)/mitogen-activated protein kinase (MAPK) pathway. This finally induces the phosphorylation and thereby activation of the cyclic adenosine monophosphate (cAMP)-response element-binding protein (CREB). CREB in turn triggers the expression of survival promoting genes within the SGN [16]–[18].

Another important activator for CREB-mediated neuroprotection is cAMP [19], [20]. The multifunctional second messenger cAMP promotes neuronal differentiation and survival [21], [22] as well as outgrowth, regeneration [23]–[25] and guidance of neuronal processes [26], [27]. Carefully increased concentrations of cAMP, as evoked by the application of cAMP analogues, promote the survival [28], [29] and enhance fibre elongation of SGN *in vitro* [30].

Another more clinically relevant option to increase cAMP levels in neurons is the application of specific phosphodiesterase type 4 (PDE4) inhibitors such as Rolipram [31]–[34]. So far, several studies have demonstrated neuroprotective [35]–[39] and anti-inflammatory [40], [41] effects of Rolipram after lesions of the central nervous system. Additionally, neuroregeneration and axonal outgrowth can be enhanced by Rolipram application [42]–[45]. Different studies reported that its beneficial effects can be enhanced when applied in combination with other protective factors or substances [42], [46], [47]. In order to exert its neuroprotective effect, Rolipram increases the level of intracellular cAMP [48].

As recently demonstrated by Xu et al., 2012 [30], the beneficial effect of intracellular cAMP on SGN critically depends on low cAMP concentrations. A previous study of our group demonstrated a protective effect on SGN *in vitro* only if Rolipram was delivered encapsulated in lipid nanocapsules [49]. However, this neuroprotective effect was not observed after treatment with pure Rolipram [49]. One explanation could be that the used Rolipram concentration induced an increase of cAMP too high to promote the protective effect demonstrated by cAMP analogues [28]–[30].

Therefore, the aim of the present study was to clarify if a Rolipram-induced increase of cAMP can be clinically relevant for the protection of SGN. To avoid ineffective high concentrations of cAMP, we tested the impact of Rolipram on dissociated SGN with focus on a lower concentration range than used by Meyer et al., 2012 [49]. We investigated the significance of single Rolipram application and co-application of Rolipram and BDNF. Our results showed that the application of Rolipram improved the survival of SGN *in vitro* when applied at a concentration of 0.1 nM. Furthermore, we were able to demonstrate that co-application of Rolipram and BDNF strongly enhances the survival promoting effect of BDNF and increases the expression or release of endogenous BDNF in different cell types of the spiral ganglion *in vitro*.

## Materials and Methods

### Ethics statement

All experiments were carried out in accordance with the institutional guidelines for animal welfare of Hannover Medical School following the standards described by the German animal protection law (*Tierschutzgesetz*). The mere killing of rats for tissue analysis is registered with the local authorities (*Zentrales Tierlaboratorium*, Hannover Medical School) and reported on a regular basis as demanded by law but needs no further approval if no other treatment is applied before sacrifice (§4-2013/44). Neonatal Sprague-Dawley rats were rapidly decapitated.

### Animals and primary spiral ganglion cell culture

For the primary spiral ganglion cell culture, explants were isolated from neonatal Sprague-Dawley rats (postnatal day 3–5). For each experimental setting, about 18 animals (36 cochleae) were used for the isolation of spiral ganglion cells.

Rats were sacrificed by rapid decapitation. The dissection of the cochleae and the enzymatic and mechanical dissociation of the spiral ganglion were performed as previously described [10], [50]. The number of viable cells was determined using a Neubauer cytometer and trypan blue staining. Dissociated cells of the spiral ganglion were seeded at defined densities in either 96-multiwell plates (1×10^4^ cells: determination of the survival rate and for single immunofluorescence analysis; 2×10^4^ cells: ELISA) or on coverslips (2×10^4^ cells; diameter 10 mm, Karl Hecht GmbH&Co KG, Sondheim: double-immunofluorescence stainings). Prior to cell seeding, the used plates and coverslips were coated with poly D/L-ornithine (Sigma-Aldrich, St.Louis) and laminin (Life Technologies, Carlsbad) as described in detail by Wefstaedt et al., 2005 [50]. The spiral ganglion cells were cultivated for 48 h at 37°C, 5% CO_2_ in a humidified atmosphere in serum free-medium (Dulbecco's modified Eagle's medium; Life Technologies, Carlsbad): determination of the neuronal survival rate; Panserin401 (PAN Biotech, Aidenbach): immunofluorescence and ELISA. Both media were supplemented with HEPES (25 mM; Life Technologies, Carlsbad), glucose (6 mg/ml; Braun AG, Melsungen), penicillin (30 U/ml; Grünenthal GmbH, Aachen), N2 supplement (3 μl/ml; Life Technologies, Carlsbad) and insulin (5 μg/ml; Sigma-Aldrich, St.Louis).

Rolipram (TOCRIS Bioscience, Bristol) and/or BDNF (50 ng/ml; Life Technologies, Carlsbad) were added to the medium respectively. The effect of Rolipram was tested at various concentrations (0.1, 1, 3, 10 nM) alone and in combination with BDNF (50 ng/ml). Treatment with Rolipram and/or BDNF was performed for the whole cultivation period (48 h) or for 30 minutes (min) for the determination of activated ERK and CREB. In the latter case, Rolipram, BDNF or both were added to the medium of pre-cultivated (either for 47 h or 30 min) spiral ganglion cells. After further 30 min of cultivation, the cells were fixed with acetone/methanol (1∶1) or paraformaldehyd (4%) solution. In all experiments, we included a negative control (spiral ganglion cells cultivated in serum-free medium) and a control of the seeding density after 4 h of cultivation.

### Survival rate of SGN

The cultures obtained from the dissociated spiral ganglion are mixed cultures containing neurons, fibroblasts, glia and satellite cells. In order to calculate neuronal survival rates, a neuron-specific staining was utilized to identify and discriminate SGN from the superior number of the other cell types. The spiral ganglion cells were fixed with a 1∶1 acetone/methanol solution (Merck KGaA, Darmstadt) in phosphate-buffered saline (PBS; Life Technologies, Carlsbad) for 10 min. After fixation, cells were washed with PBS and subsequently incubated with a monoclonal mouse 200-kD neurofilament antibody (clone RT97; Leica Biosystems, Wetzlar; 1∶500 diluted in 1.5% normal horse serum (Vector Laboratories Inc., Burlingame) in PBS) for 1 h at 37°C. After rinsing for several times with PBS, the cells were incubated for 30 min at room temperature (RT) with a secondary biotinylated anti-mouse antibody (Vector Lab, Burlingame; 1∶2000 diluted in 1.5% normal horse serum in PBS). After washing with PBS, cells reacted with ABC complex solution (Vectastain Elite ABC-Kit; Vector Laboratories Inc., Burlingame) according to the manufacturer's protocol. The staining was visualized using diaminobenzidine (Peroxidase Substrate Kit DAB; Vector Laboratories Inc., Burlingame). Controls were performed by omitting the primary antibody. To determine the cell survival rate, surviving neurons of each well were counted using an inverted microscope (IX 71, Olympus, Tokio) equipped with a mono-coloured camera (F-View, SIS) and imaging software (Analysis Version 3.2, SIS). Surviving neurons are defined as neurofilament-positive cells that exhibit a neurite outgrowth of three neuron cell soma diameters or greater in length [51]. The survival rate was calculated by relating the number of survived neurons to the mean seeding density of the respective plate.

### Immunocytochemistry and image acquisition

For immunocytochemistry, cells were fixed for 10 min in 4% paraformaldehyd (Merck KGaA, Darmstadt) in PBS. After rinsing the cells several times in 0.3% TritonX-100 (Sigma-Aldrich, St.Louis)-containing PBS (0.3% PBST), cells were blocked with 0.3% PBST containing 3% bovine serum albumin (Sigma-Aldrich, St. Louis) and incubated with primary antibodies at 4°C overnight. A list of used primary antibodies and appropriate dilutions is given in Table 1. Cells were washed with 0.3% PBST and subsequently incubated in RT with secondary antibodies (2 h, 1∶500 dilution) conjugated to Alexa Fluor 546, 488 (Life Technologies, Carlsbad) or Cy3 (Jackson Immunoresearch, Newmarket). Primary and secondary antibodies were diluted in blocking solution. Experiments, in which primary antibodies were omitted, were performed to control for non-specific binding of secondary antibodies. Finally, cells were rinsed with PBS and mounted in DAPI-containing antifade reagent (ProLong Gold; Life Technologies, Carlsbad). Fluorescence acquisition of single stainings was performed with an IX 71 Olympus microscope as described above. Double staining experiments were evaluated using an inverted LEICA DM IRB confocal microscope (Leica Biosystems, Wetzlar). Leica TCS SL image acquisition software was used to adjust offset and gain of the channels. Scanning was performed with a 40x/1.25 plan apochromat objective at a resolution of 1024×1024 pixels. Scanning of the different channels was performed separately. Images are presented as maximum projections of z-stacks of 2–3 μm thickness (z-axis increment 0.2 μm). Images were superimposed and slightly adjusted for brightness and contrast in Photoshop CS5 (Adobe Systems, San Jose).

**Table 1:** Primary antibodies and their dilutions used for immunofluorescence analysis.

Antibody	Antigen	Host, monoclonal or polyclonal, dilution	Manufacturer; catalog or log number.
Neuofilament	Cow, 200 kD neurofilament heavy chain, full length native protein	Chicken, polyclonal, 1∶500	Abcam plc, Cambridge; ab4680
pCREB	Synthetic phosphopeptide corresponding to residues surrounding SER133 of human CREB	Rabbit, monoclonal, 1∶600	Cell Signaling Technology Inc., Danvers, Massachusetts; 9198
pERK	Synthetic phosphopeptide corresponding to residues surrounding Thr202/Tyr204 of human p44 MAP kinase	Rabbit, monoclonal, 1∶100	Cell Signaling Technology Inc., Danvers, Massachusetts, 9198
Vimentin	Purified vimentin from porcine eye lens	Mouse, monoclonal, 1∶100	DAKO Deutschland GmbH, Hamburg, Germany; M0725

### Enzyme-linked immunosorbent assay (ELISA)

To investigate if the effect of Rolipram is connected to the endogenous BDNF expression and release, we performed ELISA measurements after treatment (for 48 h or 30 min) with Rolipram, BDNF or BDNF and Rolipram. Therefore, spiral ganglion cells were seeded at a density of 2×10^4^ cells in 96-multiwell plates. The BDNF concentration in the medium was determined after a cultivation period of 48 h using a BDNF-ELISA kit (Boster biological technology Co., Ltd, Fremont). Supernatants of the same conditions (at least 3 repetitions per plate) were pooled for each plate to obtain adequate amounts of raw material. The BDNF-ELISA kit was used in accordance to the manufacturer's recommendations. Briefly, standard and samples were diluted in sample dilution buffer and mixed. 100 μl of each sample was added to a well of the pre-coated well-plate and incubated for 90 min. The plate content was discarded. Subsequently, a working dilution containing biotinylated anti-BDNF antibody was added. After an incubation step of 60 min, the wells were washed with 0.01 M PBS. Thereafter, the wells were incubated with ABC working solution for 30 min. After several washing steps, 3, 3′, 5, 5′-tetramethylbenzidine colour developing solution was added to the wells followed by a final incubation step. The colour change was stopped after 20–40 min with TMB stop solution. All incubation steps were performed at 37°C. Absorbance was measured at 450 nm using a Multikan Ascent plate reader (Thermo Scientific Inc., Waltham).

### Statistical analysis

Statistical analysis was performed with Prism 5 (GraphPad, La Jolla). All results were validated by using one-way ANOVA followed by the Tukey's post test. P values of less than 0.05 were considered to be statistically significant. All quantitative data represent the means of at least three independent approaches (N), including at least triplicates of each sample (n). Error bars in the figures indicate the standard error of the mean. Levels of significance are indicated as follows: *p<0.05; **p<0.01; ***p<0.001.

## Results

### Survival rate of SGN after Rolipram treatment

To examine its effect on the first auditory neurons, spiral ganglion cells of neonatal rats were isolated for cultivation with Rolipram at different concentrations in single- and co-treatment experiments with BDNF. After a cultivation period of 48 h, the neuronal preservation was determined by calculating the survival rate of SGN after neuron-specific staining.

**Figure 1:**
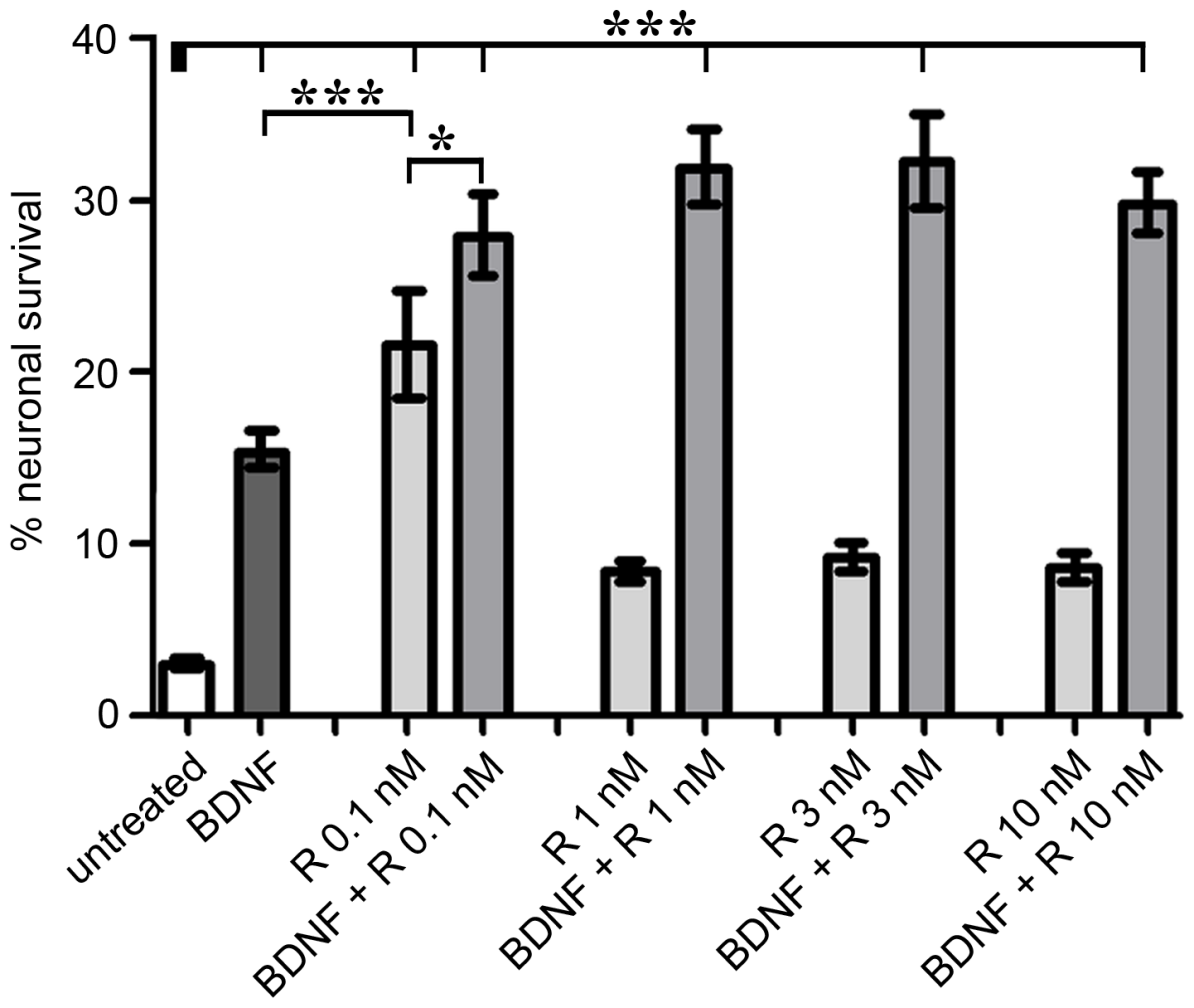
Rolipram treatment alone and in combination with BDNF improved the survival of SGN. The neuronal survival was assessed by the amount of surviving neurons after a cultivation period of 48(% neuronal survival). Rolipram (R) applied at a concentration of 0.1 nM enhanced the survival of SGN after serum deprivation and resulted in a significant higher survival rate when compared to the untreated group and to the positive control (treated with BDNF [50 ng/ml]). Elevated Rolipram concentrations (1, 3, 10 nM) did not result in an increased neuronal survival compared to the untreated control group. Co-treatment with Rolipram and BDNF caused an additional increase of neuronal survival independently of varying Rolipram concentrations. Values are given as mean ± SEM; N = 4; n = 3; One-way ANOVA with Tukey's multiple post hoc test was used to compare means: *p<0.05; **p<0.01; ***p<0.001. Reference of the significance is marked by the thick bar. N quotes the number of independent experiments; n gives the number of repetitions of each condition within one experiment.

The survival of SGN after the application of different Rolipram concentrations was compared to each other as well as to control groups represented by spiral ganglion cultures cultivated in serum-free medium without the addition of any growth factors or with BDNF (Fig. 1). Consistent with previous studies [11], [52], cells treated with BDNF showed a significantly increased survival of SGN (p<0.001) when compared to control cells.

The neuronal survival rate of Rolipram-treated cells differed in dependency of the applied Rolipram concentration: when provided at a low concentration of 0.1 nM, the survival rate of isolated SGN increased in comparison to the untreated control group (p<0.001). Application of Rolipram in this concentration was even more effective in improving neuronal survival than BDNF (p<0.001). By contrast, spiral ganglion cultures subjected to higher Rolipram concentrations (1, 3, 10 nM) revealed no improved neuronal survival in comparison to the untreated control and thereby failed to support the auditory neurons.

Furthermore, we analysed the effect of Rolipram in co-treatment experiments with BDNF. The combination of different Rolipram concentrations and BDNF led to an improved preservation of SGN. The survival rates of cultures treated with both Rolipram and BDNF were statistically higher in comparison to the untreated control (p<0.001) as well as to cultures treated with BDNF (p<0.001) or with Rolipram in the survival promoting concentration of 0.1 nM (p<0.05). Interestingly, the effective neuroprotection mediated by the combination of Rolipram and BDNF was not attenuated by increasing Rolipram concentrations as this was the case in the single-treatment approaches.

Due to the fact that 0.1 nM was the most suited concentration for the protection of SGN in our single application experiments, we exclusively used this concentration for the following investigations.

### Activation of ERK and CREB in different cell types of the spiral ganglion by application of Rolipram and BDNF

**Figure 2:**
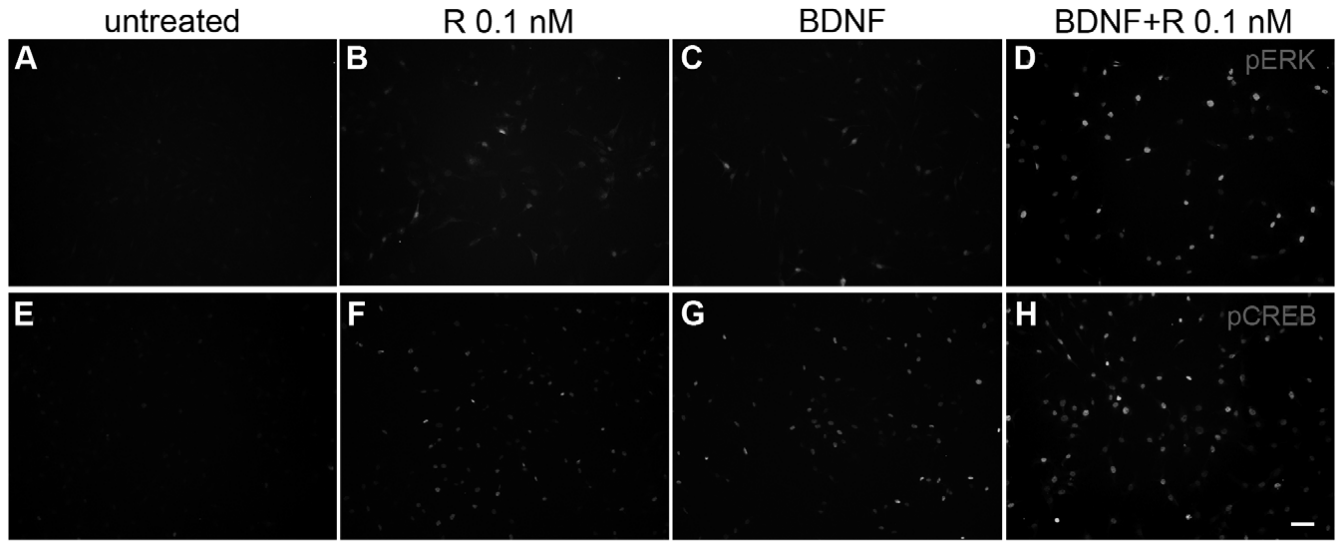
Rolipram and BDNF application induced the activation of ERK and CREB. Immunofluorescence staining of activated ERK (pERK; A–D) and activated CREB (pCREB; E–H) in spiral ganglion cell cultures incubated in medium for 48 h (A, E) or exposed to Rolipram (R; B, F) or BDNF (C, G) for 30 min after a pre-incubation of (47 h, 30 min) in medium. D, H shows the pERK and pCREB immunoreactivity in cells pre-treated with BDNF (47 h, 30 min) and additionally exposed to Rolipram for 30 min (D, H). Photographs were taken under the same conditions in which exposure time and intensity was adjusted to the untreated control group (A, E). Application of Rolipram (B, F) or BDNF (C, G) induced the activation of ERK and CREB. Stronger pERK and pCREB immunoreactivity was observed after co-treatment with Rolipram and BDNF (D, H). BDNF: brain-derived neurotrophic factor; R: Rolipram. Scale bar: 50 μm (A–H).

The influence of Rolipram and BDNF applications on intracellular signaling pathways was also examined. Hence, we investigated two major components of Rolipram- or BDNF-mediated intracellular cascades by immunocytochemistry in primary spiral ganglion cells cultured either with Rolipram, BDNF or both substances (Fig. 2).

In these analyses, we investigated the transcription factor CREB as a common target in pathways induced by both Rolipram and BDNF [44], [53] and the protein kinase ERK, which is a major effector within the BDNF signaling pathway [15]. In case that both proteins are ubiquitously expressed, we used antibodies that specifically detect the phosphorylated and thereby activated form of CREB (pCREB) and ERK (pERK1/2).

It was previously shown that an induced activation of ERK and CREB by specific substances is a dynamic and time-dependent process, which can effectively be detected after a short exposed time of 15–60 min [54], [55]. Therefore, the following experiments were performed with pre-cultivated (48 h) spiral ganglion cells, incubated for 30 min either with BDNF (used for cells pre-cultivated in medium) or Rolipram (used for cells pre-cultivated in medium for single Rolipram application or pre-treated with BDNF for co-treatment experiments). Figure 2 shows the detection of pERK1/2 and pCREB in untreated spiral ganglion cells (Fig. 2A, E) and in cells treated with Rolipram (Fig. 2B, F), BDNF (Fig. 2C, G) or both (Fig. 2D, H). Untreated cultures showed a marginal immunofluorescence of both proteins present in all cells, indicating a basic activation of both proteins under normal culture conditions (Fig. 2A, B). In Rolipram- or BDNF-treated cultures, the immunoreactivity of both factors was more prominent in a defined number of cells (Fig. 2C, D, F, G). The immunoreactivity of ERK (Fig. 2D) and CREB (Fig. 2H) was even more enhanced in conditions treated with both BDNF and Rolipram. This was obvious not only due to the number of stained cells but also due to the enhanced fluorescence signal. Nevertheless, the activation of ERK and CREB was restricted to a distinct number of spiral ganglion cells.

Thus, the cell type or cell types that responded to the co-application of Rolipram and BDNF with an excessive activation of ERK and CREB were identified using immunocytochemistry. The spiral ganglion comprises two types of neurons (type 1 and type 2) and different non-neuronal, supporting cells predominantly represented by glial cells and fibroblasts. Neurons can be identified by immunostainings with anti-neurofilament antibodies, glial cells and fibroblasts can be marked with antibodies against the intermediate filament vimentin.

**Figure 3:**
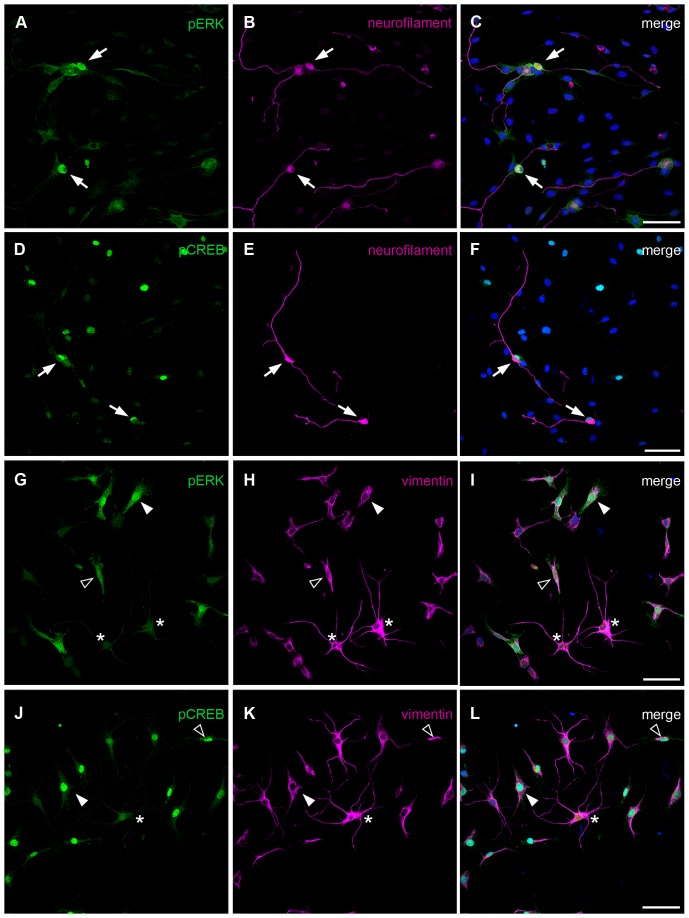
ERK and CREB activation in neurons and distinct vimentin-positive cells by Rolipram/BDNF co-treatment. Projections of collapsed confocal scans showed double-stainings with pERK (A, G) or pCREB (D, J) antibodies and antibodies directed against neuron-specific 200 kDa neurofilament (B, E) or vimentin (H, K) in spiral ganglion cell cultures pre-treated with BDNF (47 h, 30 min) and exposed to Rolipram for 30 min. Nuclei were stained with DAPI. Arrows point to an intense labelling of pERK (C, F) or pCREB (I, L) in the somata of stained SGN. Additionally, pERK (C, F) and pCREB (I, J) immunosignals were present in distinct vimentin-positive cells with a flattened (filled arrowhead) or spindle-shaped (unfilled arrowhead) morphology. Asterisks mark cells with a star-like morphology with a weak reactivity for pERK. Scale bar: 70 μm in C, 75 μm in F, I, L).

For the identification of the Rolipram-sensitive cell source, we combined pERK and pCREB immunocytochemistry with both neuronal and non-neuronal markers (Fig. 3). Double-labelling experiments with neurofilament and pERK or pCREB respectively showed the activation of the kinase and of the transcription factor in the stained SGN (Fig. 3 A–F, pointed by arrows). Additionally, the activation of ERK and CREB was demonstrated in at least one other non-neuronal cell type of the spiral ganglion labelled with vimentin (Fig. 3 G–L). The appearance of these cells matched the morphological description of fibroblasts with a flat and expanded cell body (Fig. 3 G–L; pointed by white arrowheads) or less often with a bipolar structure and an elongated spindle shaped soma (Fig. 3 G–L; pointed by the filled arrowheads). By contrast, pERK- and pCREB-negative cells often exhibit a star-like morphology (Fig. 3 G–L) that might be referred to astro-glial cells. Similar was observed in cultures after treatment with either Rolipram or BDNF (data not shown).

### Increased endogenous release of BDNF from BDNF-preconditioned spiral ganglion cells after treatment with Rolipram

**Figure 4:**
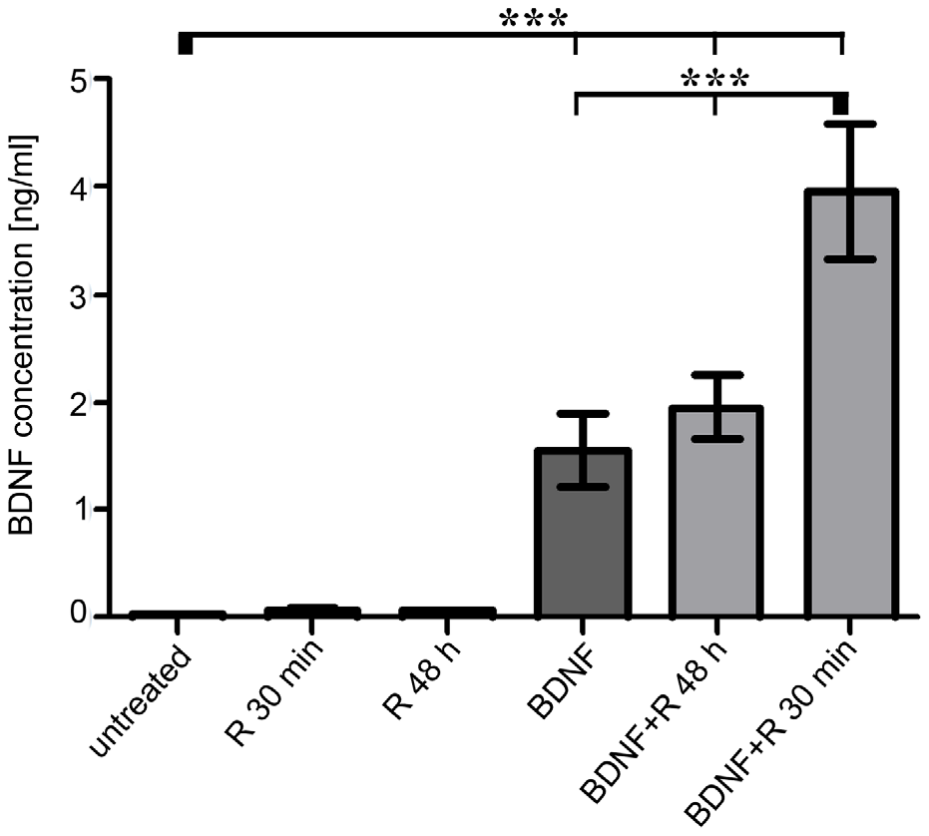
BDNF release from spiral ganglion cells is increased after co-treatment with Rolipram and BDNF. The concentration of released BDNF from spiral ganglion cells after treatment with Rolipram (R; 48 h and 30 min), BDNF (48 h) or both substances (BDNF 47R 30 min) was measured by ELISA. The supernatant of cells treated with BDNF alone or in combination with Rolipram contained significant higher concentrations of BDNF in comparison to supernatants from untreated cultures or from cells treated only with Rolipram. Application of Rolipram for 30 min to BDNF pre-treated cells resulted in a strong increase of BDNF in the supernatant. All values are given as mean ± SEM. N = 3; n = 3 except for the untreated and Rolipram 48 h group: N = 2, n = 3; One-way ANOVA with Tukey's multiple post hoc test was used to compare means: *p = 0.05; **p = 0.01; ***p = 0.001. Reference of the significance is marked by the thick bar. N gives the number of independent approaches; n gives the number of samples per approach.

Our immunocytological results clearly showed that Rolipram and BDNF led to an increased activation of CREB (Fig. 2, Fig. 3). CREB, acting as a transcription factor, is involved in the expression of various neuroprotective-acting genes [19]. In correlation with the protection of SGN, one very interesting target of CREB is BDNF [35], [56]. Therefore, we investigated if the observed protection of SGN by Rolipram and BDNF (Fig. 1) is mediated by an induced increase of the endogenous BDNF expression. Hence, supernatants from cultured spiral ganglion cells were collected after 48 h of cultivation for quantification of the BDNF amount by ELISA. The BDNF concentration in medium obtained from cell cultures treated with BDNF, Rolipram or both for 48 h or 30 min was analyzed (Fig. 4). Additionally, the supernatant from untreated spiral ganglion cells was assayed and served as control.

Single treatment with Rolipram did not significantly influence the release of BDNF from SGN, neither when applied for the whole cultivation period of 48 h nor when exposed for 30 min. Nevertheless, the measured mean concentration of BDNF was elevated by trend from 0.032 ng/ml (supernatant of untreated control cells) to 0.051 ng/ml (incubation with Rolipram for 48 h) and further to 0.071 ng/ml (cells exposed to Rolipram for 30 min).

By contrast, the application of recombinant BDNF resulted in an increased BDNF concentration in the supernatants: after a cultivation period of 30 min, the concentration of BDNF was added up to 40.04 ng/ml (data not shown). In case of externally applied BDNF, we cannot distinguish between recombinant and endogenous BDNF: even so, we determined the BDNF-concentration in supernatants of cultures exposed to recombinant BDNF for 48 h. Such cultures contained a nearly fifty-fold BDNF (1.56 ng/ml) concentration when compared to the untreated control group (p<0.001).

Co-application of BDNF with Rolipram intensified the release of BDNF that was induced by the application of recombinant BDNF alone: after a short incubation with Rolipram for 30 min of cultures pre-treatment with recombinant BDNF for 48 h, the measured amount of BDNF in the cultivation medium increased by more than 150% (up to 3.95 ng/ml; p<0.001). By contrast, incubation of cells with BDNF and Rolipram for 48 h did not result in a statistically higher release of BDNF. Here, the BDNF concentration stepped up solely by trend from 1.56 ng/ml (BDNF alone for 48 h) to 1.953 ng/ml (BDNF+Rolipram both for 48 h).

## Discussion

After damage of the hair cells, the preservation of residual SGN and the regeneration of their degenerated processes are necessary procedures to improve hearing sensation with a cochlear implant in affected people. The current study demonstrated the therapeutic capacity and required conditions of Rolipram to support neonatal SGN *in vitro* under serum deprived conditions and clearly demonstrates the necessity of the adequate dosage.

### Rolipram protects SGN after serum deprivation and its concentration is critical for the neuroprotective effect

Our results clearly demonstrated that Rolipram applied to cultured spiral ganglion cells increased the neuronal survival after a cultivation period of 48 h. The protection of Rolipram was more potent than the effects evoked by BDNF. This neuroprotective effect of Rolipram was restricted to the low concentration of 0.1 nM. By contrast, the combined application of Rolipram alongside with BDNF increased the survival-promoting capacity of Rolipram significantly by 11% independently of its applied concentration.

Rolipram acts by inhibition of the PDE4 and thereby indirectly by the intracellular increase of cAMP. As described for cAMP [30], we here show that the concentration of Rolipram is critical for the protective effect. The biphasic effect of cAMP described for neuritogenesis [30] may also apply for neuronal survival: increasing survival at low concentrations and vice versa as demonstrated by the results of the present study. Thus, increase of cAMP above a critical point -as possibly induced by Rolipram if applied at too high concentrations- might turn into reverse and unwanted effects.

A previous study from Meyer et al., 2012 [49] reported that a protective effect of Rolipram on cultured SGN was exclusively obtained when it was provided encapsulated in lipid nanocapsules. They hypothesized that the translocation of Rolipram into the cell was only effective enough to mediate the protective effect when mediated by lipid nanocapsules. Based on our results and several other studies demonstrating the protection of neurons by different treatment methods with Rolipram [35]–[39], we suggest that the concentration of Rolipram applied by Meyer et al., 2012 [49] was too high to increase neuronal survival, whereas the delivery via the lipid nanocapsules allowed a lower and thereby efficient Rolipram concentration for the induction of the protective effects.

### The neuroprotective effect of Rolipram and BDNF is mediated by different intracellular signalling pathways

The protective effect of Rolipram was by 35% stronger than the effect of the neurotrophic factor BDNF. Though both substances lead to an increased activation of ERK and CREB, we assume that Rolipram and BDNF activate different intracellular signalling pathways with distinct neuroprotective potencies. Our results demonstrated that the Rolipram-induced intracellular pathways are more effective in promoting the survival of neonatal SGN.

This assumption was additionally supported by the ELISA measurements that demonstrated the involvement of endogenous BDNF only in the cultures pre-conditioned with BDNF and treated with Rolipram. By contrast, the more potent protection of SGN by Rolipram proceeds independently of BDNF.

Rolipram specifically inhibits the activation of the PDE4 and thereby decreases the degradation of the second messenger cAMP that triggers the protective effect of Rolipram primarily via an activation of the cAMP-dependent PKA [31]–[34]. Two different mechanisms have been proposed so far for the activation of PKA leading to the protection of SGN.

1. Induction of gene expression by activation of CREB: PKA is able to activate CREB directly by translocating into the nucleus or indirectly via the activation of ERK [21], [57]. Our results corroborate other studies demonstrating that the beneficial effect of Rolipram is mediated by the activation of ERK and CREB [39], [48], [58]. Finally, CREB regulates the expression of several important pro-survival genes, which might be up-regulated by the Rolipram-induced activation of CREB [19].
2. Modification of the pro-apoptotic protein BAD: cyclic AMP and PKA are important modulators of anti-apoptotic signalling mechanisms resulting in cell protection by post-translational modifications of the pro-apoptotic protein BAD [59]–[61]. Bok et al., 2003 [28] showed that such modifications may also account for an increased survival of SGN.

According to this, we assume that the potent Rolipram-induced neuroprotection is based on the activation of different parallel pathways that influence post-translation processes as well as specific gene-targeting via CREB activation.

The intracellular signalling pathway of BDNF is well described. BDNF acts by the activation of TrkB receptors that are also present in the different cell types of the spiral ganglion [15]. Activated TrkB receptors signal via various downstream pathways that facilitate the survival of the cell. For example, by activating the phosphatidylinositol-3-kinase, it finally leads to the phosphorylation and thereby inactivation of pro-apoptotic targets [62]. Another important downstream pathway of TrkB is the ERK-MAPK pathway resulting in the activation of CREB [53]. The protective effect of this pathway is mediated by the CREB-dependent activation of the transcription of different genes that prolong the cellular survival [19].

### Activation of CREB induced only by Rolipram does not increase endogenous expression of BDNF

Interestingly, if Rolipram is applied alone, it is sufficient to promote survival and to activate CREB but not to induce release of endogenous BDNF as demonstrated by the ELISA results. By contrast, the activation of CREB induced by the combined application of recombinant BDNF and Rolipram additionally triggers the endogenous expression of BDNF. These results are in line with several studies describing that CREB also induces the expression of BDNF in general [16], [53], [56] and specifically also in SGN [63]. The expression of BDNF upon activation of CREB may be due to the presence of specific transcriptional co-activators that may be recruited by the activation of the Trk receptor-mediated pathway.

Different co-factors in the diverse signalling pathways that result in the activation of CREB are important determinants of the CREB-dependent gene targeting [64]. Based on the recruitment of such co-factors, the expression of specific genes may be induced by CREB [64]. Besides this, Rolipram may also activate protective pathways independent from BDNF yielding to a maximum of protection as demonstrated by the survival rate. As demonstrated, the increased neuronal survival after combined application of Rolipram and BDNF was interestingly not affected when Rolipram was applied in higher concentrations, whereas the neuroprotection by single Rolipram application was strictly limited to a low concentration of 0.1 nM (Fig. 1). One explanation for this phenomenon might be the distinct roles of the intracellular co-factors that are activated either by Rolipram or by BDNF.

As described above, higher concentrations of Rolipram may increase intracellular cAMP leading to apoptosis. The role of cAMP in the activation of apoptosis-mediating signalling pathways has been described recently [65]. It is also known that SGN express cAMP-sensitive cationic channels [66]. Thus, we may assume that constitutive opening of such channels with increased Ca^2+^-influx may account for apoptosis and the lowering of survival rates after the application of higher concentrations of Rolipram. However, a simultaneous activation of the TrkB-MAPK-pathway by the co-application of recombinant BDNF counteracts the toxicity of increased intracellular cAMP. This may either be due to the endogenous release of BDNF or due to the recruitment and inactivation of co-factors that account for cAMP-mediated cytotoxicity.

The method used to quantify BDNF in our culture supernatants does not distinguish between recombinant and endogenous BDNF. Therefore, cultures treated only with recombinant BDNF may also contain endogenously released BDNF, whereas after treatment with Rolipram alone we did not detect relevant levels of endogenous BDNF. As described by Soto et al., 2006 [57], activation of PKA enables a direct activation of CREB. In addition, the transcriptional potential of CREB can be modulated epigenetically. For example, methylation of cytosine within CRE sites inhibits binding of CREB to DNA [67] and thereby CRE-dependent transcription. This process can be regulated dynamically and seems responsible for the inducible BDNF-expression that has been described in the central nervous system [56]. It is not clear how these epigenetic changes are induced and if they may occur upon cAMP-PKA-induced CREB activation without the co-activation by the Trk-receptor-mediated pathway.

### Neuroprotection by endogenous BDNF

As published previously [10], our results confirm that endogenous BDNF is much more efficient in supporting the vitality of the neurons of the spiral ganglion than the application of the recombinant protein. The latter is usually administered at a concentration of 50 ng/ml. In our experiments, we did not distinguish between recombinant and endogenous BDNF. Thus, the BDNF concentration measured 48 h after the application of recombinant BDNF (0.07815 pg/cell, 1.563 ng/ml) might also contain residual recombinant BDNF. However, the 2.5-fold increase of the BDNF-concentration after application of Rolipram to BDNF pre-conditioned cultures (B48+R30: 0.1974 pg/cell, 3.948 ng/ml) indicates that at least more than half of the measured BDNF (0.11925 pg/cell, 2.385 ng/ml) might be of endogenous origin. In our previous study [10], we measured a similar endogenous BDNF-release after 7 days of cultivation (0.116 pg/cell) in immortalized fibroblasts genetically modified to express BDNF. Thus, in relation to this artificial overexpression system, the addition of Rolipram to BDNF-preconditioned cultures of spiral ganglion cells accounts for a high release of endogenous BDNF.

### The protective effects were mediated by different cell types of the spiral ganglion

Based on the herein presented results, the effect of Rolipram is not restricted to the neuronal cells within the spiral ganglion. Other cell types, such as fibroblasts, satellite or Schwann cells are also activated after administration of Rolipram. The anti-apoptotic effect of Rolipram on fibroblasts has already been described [68]. Possibly, Rolipram might lead to an up-regulation of fibroblast growth factor by fibroblast and glial cells that is also known to exert beneficial effects on SGN [9], [69]–[71]. In addition, the effect of BDNF is not restricted to the neurons and co-administration of recombinant BDNF with Rolipram might induce the endogenous BDNF expression in the supporting cells of the spiral ganglion. This is in corroboration with earlier studies that have demonstrated the endogenous BDNF expression in astrocytes and activated microglial cells [72], [73].

Considering the fact that the neuronal population in the spiral ganglion comprises of much lower numbers when compared to the fibroblasts and glial cells, it is of advantage that the endogenous protective mechanisms induced by a pharmacological substance are not limited to the neurons. Moreover, the activation of the endogenous natural protective mechanisms of the supporting cells is favourable.

In summary, our *in vitro* study identified Rolipram as a promising candidate for the protection and the enhancement of the survival of SGN. In the *in vitro* model, our results demonstrated that the application of Rolipram after pre-conditional treatment with BDNF (that can be applied intraoperatively and locally as a single-bolus to the cochlea) acted synergistically by increasing the auto-/paracrine survival-promoting effects of different cell types in the neonatal spiral ganglion and by enhancing significantly the neuronal survival rate. Distinct properties of Rolipram, i.e. low molecular weight and the ability to pass the blood-brain barrier [38], are beneficial for several application forms such as subcutaneous or oral administration [25]. The strong stability under physiological conditions provides also the possibility for a long-term application via osmotic pumps or drug reservoirs that are well suited for a constant supply of the spiral ganglion after insertion of a cochlear implant. Here, Rolipram was used as a representative of its class, the PDE4 inhibitors. PDE4 inhibitors can be used for long-term application as has been proven already by the clinical application of Roflumilast for the treatment of chronic obstructive lung disease with few unthreatening adverse effects such as diarrhea, nausea and weight loss [74].

Interestingly, also a regenerative [25], [42], [43] and anti-inflammatory [40], [41], [75] potential is described for Rolipram. Therefore, we suggest that besides the capability of improving the vitality of SGN after insertion of a cochlear implant, Rolipram might also be able to reduce the inflammation due to the insertion process and potentially mediate a regenerative effect on the deprived processes of the SGN by initiating their outgrowth towards the implant electrode. The latter have to be explored in future studies. Furthermore, by the inhibition of possible intracellular key adaptor and effector proteins, distinct intracellular cascades of BDNF as well as their interrelation to Rolipram may be identified *in vitro*. One limitation of this study is the proof of effect on neonatal and cultured SGN. Since this artificial environment does not represent the conditions *in situ*, it has to be considered that the effects of Rolipram in the adult system and *in vivo* may differ from the herein presented results. Thus, *in vivo* experiments will be important and necessary to verify the presented data in the natural system and to identify application modalities for a prolonged release of endogenous BDNF.
